# The evolution of lossy compression

**DOI:** 10.1098/rsif.2017.0166

**Published:** 2017-05-10

**Authors:** Sarah E. Marzen, Simon DeDeo

**Affiliations:** 1 Physics of Living Systems, Department of Physics, Massachusetts Institute of Technology, 77 Massachusetts Avenue, Cambridge, MA 02139, USA; 2 Redwood Center for Theoretical Neuroscience, and Department of Physics, University of California at Berkeley, Berkeley, CA 94720, USA; 3 Department of Social and Decision Sciences, Carnegie Mellon University, 5000 Forbes Avenue, BP 208, Pittsburgh, PA 15213, USA; 4 Santa Fe Institute, 1399 Hyde Park Road, Santa Fe, NM 87501, USA

**Keywords:** lossy compression, rate–distortion, information theory, perception, signalling, neuroscience

## Abstract

In complex environments, there are costs to both ignorance and perception. An organism needs to track fitness-relevant information about its world, but the more information it tracks, the more resources it must devote to perception. As a first step towards a general understanding of this trade-off, we use a tool from information theory, rate–distortion theory, to study large, unstructured environments with fixed, randomly drawn penalties for stimuli confusion (‘distortions’). We identify two distinct regimes for organisms in these environments: a high-fidelity regime where perceptual costs grow linearly with environmental complexity, and a low-fidelity regime where perceptual costs are, remarkably, independent of the number of environmental states. This suggests that in environments of rapidly increasing complexity, well-adapted organisms will find themselves able to make, just barely, the most subtle distinctions in their environment.

## 1. Introduction

To survive, organisms must extract useful information from their environment. This is true over an individual's lifetime, when neural spikes [1], signalling molecules [2,3] or epigenetic markers [4] encode transient features, as well as at the population level and over generational timescales, where the genome can be understood as hard-wiring facts about the environments under which it evolved [5]. Processing infrastructure may be built dynamically in response to environmental complexity [6–9], but organisms cannot track all potentially useful information because the real world is too complicated and information bottlenecks within an organism's sensory system prevent the transmission of every detail. In the face of such constraints, they can reduce resource demands by tracking a smaller number of features [10–14].

Researchers have focused on how biological sensory systems might optimize functionality given resource constraints, or, conversely, minimize the resources required to accomplish a task. In neuroscience, the efficient coding hypothesis [15] and minimal cortical wiring hypothesis [16] are two well-known and highly influential examples. Much of this work has focused on maximizing the ratio of transmitted information (usually, the mutual information between stimulus and spike train) to the energy required for such a code, e.g. [17–24].

Researchers often talk about the creation of more efficient codes that find clever ways to pass information from sensors to later stages in the brain's processing system by adapting to changes in environmental statistics [25–27] and selectively encoding meaningful features of the environment [28]. However, there is much less understanding of what happens when an organism responds to environmental complexity by discarding information, rather than finding ways to encode it more efficiently.

In this paper, we use an information-theoretic tool—rate–distortion theory—to quantify the trade-off between acquisition costs and perceptual distortion [29]. Rate–distortion allows us to talk about the extent to which an organism can save costs of transmitting a representation of their environment by selectively discarding information.

When they do such a compression, evolved organisms are expected to structure their perceptual systems to avoid dangerous confusions (not mistaking tigers for bushes) while strategically containing processing costs by allowing for ambiguity (using a single representation for both tigers and lions)—a form of *lossy* compression that avoids transmitting unnecessary and less-useful information. Our use of this paradigm connects directly to recent experimental work in the cognitive sciences that uses rate–distortion theory to study errors made in laboratory perception exercises [30].

Lions and bushes are informal examples from mammalian perception, but similar concerns apply to, for example, a cellular signalling system which might need to distinguish temperature signals from signs of low pH, while tolerating confusion of high temperature with low oxygenation. We include both low-level percepts and the higher-level concepts they create and that play a role in decision-making [31–33]. Transmission costs include error-correction and circuit redundancy necessary in noisy systems [34], as well as costs associated with more basic trade-offs associated with volume and energy resources and that appear even when internal noise is absent [16,35–37].

Our work applies both to the problem of how an organism gathers information from the environment (the problem of sensory ecology), and how that information is transferred within the organism's cognitive apparatus from one unit to another (as happens, for example, in communication between internal encoder and decoder units in what [38] describes as ‘Marr's motif’ [39]).

In §2, we briefly review rate–distortion theory and how it provides a model of the trade-offs between perception and accuracy for biological organisms. In §3, we show that this general formalism predicts the existence of two distinct regimes in which an organism can operate. In what we refer to as the ‘high-fidelity’ regime, where an organism places a premium on accurate representations, the resources an organism requires to achieve that accuracy grow in tandem with environmental complexity, potentially without bound. This is true even when the organism accepts a certain level of error. However, if the organism is able to tolerate a critical level of error in its representations, this picture changes drastically. In this ‘low-fidelity’ regime, the resources required to achieve a given perceptual accuracy are fixed even when the environment grows in complexity. In §4, we discuss the application of these results to the understanding of perceptual systems in the wild.

## 2. Theoretical framework

There are both fitness costs and fitness benefits to accurate sensory perception ([40] and references therein). It is costly to set-up the neural architectures that promote increased perceptual ability, for example, and it is also costly to use them. On the other hand, increased perceptual ability can increase fitness, by (for example) allowing the organism to avoid predators, or more efficiently gather scarce resources.

Well-adapted organisms will necessarily balance these costs and benefits, but it is not obvious how this is to be quantified. An organism that has more efficient coding mechanisms will be able to perceive more information in the environment than one that does not.

We tackle this problem in a simplified set-up previously studied in [41]. As in [42], model organisms have (i) an adaptive representation system, which we refer to as a ‘sensory codebook’, and that specifies how an environmental state affects, probabilistically, the internal state of the organism and (ii) two constants: a *rate*, *r*, at which the perceptual apparatus gains information from the environment, and a *gain*, *β*, which quantifies the costs of doing so.

While an organism can save energy and effort by devoting only limited resources to perception, it incurs costs from doing so. This is quantified using the *distortion measure* 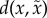, which specifies the cost incurred by an organism confusing an environmental state *x* for another state, 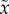.

Distortion and rate together dictate an organism's fitness. We can think (for example) of the average distortion as correlated with the energy that the organism failed to take from the environment, while rate correlates with the energy that the organism expended in making the measurements necessary to make that penalty low. Seen in this way, the organism has two competing goals, wanting to minimize both the costs of gathering information (by throwing out some of it), and the costs of acting on the information that, because it is incomplete, may occasionally be misleading.

If we consider a sensory apparatus of the brain, and fix a particular coding system, then rate *r* serves as a proxy for neuron number, so that the energy expenditure of this organism's brain is *β*^−1^*r*, where *β*^−1^ is the average rate of energy use for a single neuron [43].

In our simple model here, this enables us to make the assumption that the total cost to the organism is given by this critical quantity, *D* + *β*^−1^*r*, and that the fitness of an organism, in an evolutionary sense, is a monotonically decreasing function *f* of the organism's total cost. (In general, our results are robust to the further generalization of the fitness cost to a sum of monotonically increasing functions of both *D* and *r*; for simplicity in this paper, we consider the linear case.)

At each generation, the organism chooses its rate and gain prior to any environmental exposure [44]. The environment size *N* is chosen separately, and a new distortion measure *d* is drawn from an ensemble of *N* × *N* matrices with probability *p*(*d*). We use *ρ*(*d*) to denote the probability density function from which entries are drawn at the risk of confusion. An organism with parameters *r* and *β* has fitness *f*(*D* + *β*^−1^*r*) in that generation.

So far, we have defined (i) the costs of perception, through a rate *r* at which the organism gains information from the environment, (ii) the costs of acting in the presence of incomplete information, specified by the distortion matrix *d*, and (iii) a parameter *β* which specifies the particular way an organism balances the two costs against each other: an organism with a larger *β* finds it easier to process information.

Now, we show how both *r* and *d* can be related to a perceptual process. It is simpler to define *d* first. We say that the organism's sensory codebook maps each one of the *N* environmental states, *x*, to one of *N* sensory percepts. Further downstream in the organism's processing of this stimuli, it must then decode this sensory percept into an estimate of the true state of the environment. When the estimated environmental states are not equivalent to the true input, the organism incurs an average per-symbol cost that we assume to be given by 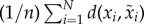, where 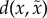 is the distortion measure defined above, *n* is the number of observations, and *x*_*i*_ the *i*th observation. We assume that there is no cost to perfectly representing an environmental state, *d*(*x*, *x*) = 0, i.e. that the distortion measure is normal. Over time, the average cost per input symbol tends to an average distortion *D*,

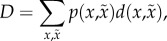
where 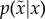 describes the probability that an environmental state *x* (drawn from the set *X*) is represented by the percept symbol 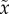 (which, for simplicity, we can also assume to be also drawn from the set *X*).

Having defined *d*, we then define *r* as the mutual information, 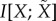 between the environment and the perceptual state. Mutual information captures the *r* of information transmission, by quantifying the drop in uncertainty about the environment from before the percept arrives to after it has been received
2.1
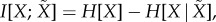
where *H*[*X*] is the entropy (uncertainty) of the environment, and 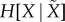 is the conditional entropy, given knowledge of the perceptual state 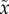.

When perception is perfect, each *x* is mapped to a unique 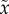 and the second term is zero: on receiving the downstream perceptual data, the organism has zero uncertainty. This means that the information about the environment has been completely transmitted to the organism. However, in the real world, constraints on the rate of information transmission will mean that only some of the information is passed along, and this means that only an approximate mapping is possible. In this case, the information transmitted is lower than what is in the environment, the second term in equation (2.1) will be greater than zero, and, as a consequence the associated codebooks will be noisy. They will occasionally map an environmental state *x* to the wrong percept (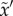, say, instead of 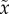), and *D* will be non-zero.

We have shown how to relate both *r* and *d* to the perceptual process, via a codebook *p*. Some codebooks, of course, are better than others, and rate–distortion focuses on those codebooks that do best given a particular hard constraint on *D* (or, equivalently, a hard constraint on *r*). The rate–distortion function *R*(*D*) defines an asymptotically achievable lower bound on the rate required to achieve a given average level of distortion
2.2
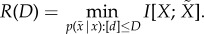
One can interchangeably speak of the distortion–rate function, the inverse of the *R*(*D*) function
2.3
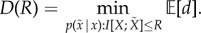
The 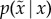 which achieve the minima provide a guide as to the kinds of confusions one would expect to see in optimal or near-optimal sensory codebooks [45].

There are intuitive reasons that one might see the rate as a natural resource cost. The most obvious, perhaps, is that, once one fixes an encoding system, the required number of sensory relay neurons required is also lower-bounded by the rate–distortion function. This bound is weakened if the neurons are not encoding sensory information combinatorially, as may happen in a compressed-sensing situation further downstream of the perceptual apparatus [38]. In the extreme case, where every distinct state is given a separate neuron then *N*_eff_ = 2^*R*(*D*)^, the (effective) number of states stored by an organism's codebook at a single time step, might be the more relevant processing cost. Increased neuron number leads to an increase in both preset and operational costs. In either case, there is evidence that an increase in neuron number yields a corresponding increase in the accuracy of the organism's internal representation of the environment [44]. Alternatively, the rate of heat dissipation needed to store and erase sensory information is at least 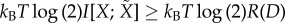 [46,47], where *T* is the ambient temperature and *k*_B_ is Boltzmann's constant. This can be viewed as a (possibly weak) lower bound on the operational cost of memory.

Rate–distortion theory is a departure from other normative efforts in that while the mutual information between stimulus and response quantifies the rate, representational accuracy is quantified by a distortion measure. Transmission rate can either be directly connected to energy or material usage, or understood more generally as a costly thing to evolve [42]. Conversely, the cost of inaccuracy has been neglected. In the past, investigators have assumed that the organism intrinsically cared about information flow [48–50], perhaps implicitly appealing to its operational definitions via the rate–distortion theorem [29], and either showed that empirical codebooks were similar to information-theoretic optimal codebooks [48,49] or inferred an effective distortion measure [30,50].

In some cases [49,51,52], the distortion measure was chosen to be a predictive informational distortion, in which sensory percepts are penalized by their inability to predict future environmental states. Here, we have introduced non-intrinsic environmental structure via a distortion measure 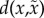 that dictates the nature of the representations that the system uses.

To calculate the rate–distortion function *R*(*D*), we find the codebook 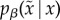 which minimizes the objective function
2.4
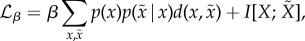
by iterating the nonlinear equation
2.5
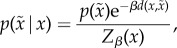
where 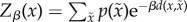 as described in [53] for 500 iterations at each *β*, or 1000 iterations at each *β* if needed. Because fitness is convex in the choice of the codebook, finding the optimal point corresponds to an unsupervised classification of sensory signals based on gradient descent. It is thus a natural task for an organism's neural system [54–57], and also corresponds to the simplest models of selection in evolutionary time [58]. From this optimal codebook, we find the corresponding expected distortion *D*_*β*_ and rate *R*_*β*_ as
2.6
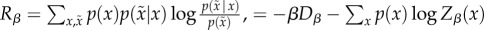
and
2.7
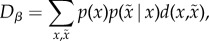
respectively. Expected distortion is at most 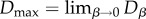. By varying *β* from 0 to ∞, we parametrically trace out the rate–distortion function *R*(*D*). In our simulations, we choose *β*'s such that the corresponding *D*_*β*_'s evenly tile the interval between 0 and 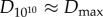. Code to perform these calculations, including the construction of the codebook that minimizes the Lagrangian in equation (2.4) given a distortion matrix, is available in [59].

In this article, we assume that the distribution over *N* possible inputs *p*(*x*) is uniform, so *p*(*x*) = 1/*N* for all *x*. We view *H*[*X*] = log *N* as a proxy for environmental complexity. In §3, we focus on the case where off-diagonal entries of 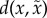 are drawn independently from some distribution *ρ*(*d*) with support on 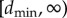, and set *d*(*x*, *x*) = 0.

Every environment has a ‘minimal confound’, defined as the minimal distortion that can arise from a miscoding of the true environmental input; mathematically, it is defined as
2.8
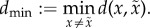
In this paper, we study exponential and lognormal distributions, shifted so that 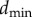 is equal to 0, 1 or 20. These provide minimal models of unstructured environments with many degrees of freedom in which some mistakes are more costly than others. The relationship between an organism's toleration of (average) distortion, and the minimal confound level, will turn out to be the dividing line between the low- and high-fidelity regimes.

## 3. Results

Evolutionary processes select organisms for higher fitness; the fitness of an organism can be thought of as its reproductive rate relative to average. A higher than average fitness will, given sufficiently faithful reproduction, translate into exponential gains for that organism and its descendants. Rather than considering absolute differences we can then focus on rank order—what changes cause the fitness to rise or fall relative to baseline.

Recall that in our simple set-up, an organism's fitness is some monotonically decreasing function *f* of the organism's total energetic cost, *D*(*r*) + *β*^−1^*r*, which has three parameters: *β* is the inverse of the average rate of energy use of a single neuron, referred to as ‘gain’; *r* is the organism's genetically determined maximal allowable rate; and *D*(*r*) is the environment-dependent distortion–rate function at rate *r*. Here, we have assumed that the sensory codebook has minimized distortion for its given rate, and so set distortion to be the distortion–rate function evaluated at *r*.

Even simple organisms, like single-cell bacteria, exist in environments in which *N* is very large, e.g. as in [48]. Practically speaking, then, we are often in the regime in which our resources are dwarfed by the environmental complexity. Naively, we might conclude that in this limit, internal representations of the environment must be quite inaccurate, i.e. 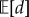 must become increasingly large. As it turns out, this intuition is half-right. Our results suggest that an organism will only operate and continue to operate at high levels of perceptual accuracy when its gain *β* grows in tandem with environmental complexity.

The minimal confound 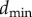 defines the boundary between two distinct regimes. In the low-fidelity regime, when 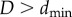, as environmental complexity grows larger and larger, the resources required to achieve a given distortion *D* asymptote to a finite constant. In the high-fidelity regime, when 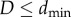, the resources required to achieve that error rate grow without bound.

**Figure 1.**
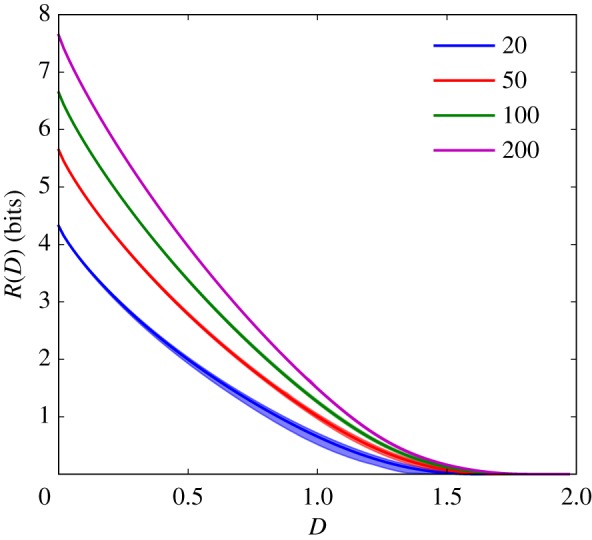
Rate–distortion functions for mean-shifted exponential *p*(*d*) for increasing *N*. Rate–distortion functions for *N* of 20 (bottom curve), 50, 100 and 200 (top curve), and 
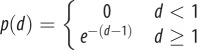
, so that *d*_min_ = 1. Shaded regions indicate 68% confidence intervals calculated by bootstrapping from 50 samples, and lines within those shaded regions are the means. At *N* ≥ 100, rate–distortion functions for different draws from the ensemble are nearly identical. As *N* grows, *R*(*D*) asymptotes to a constant when *D* > 1, but grows without bound when *D* < 1; visually, this means that, reading vertically the *R*(*D*) curves for increasing *N* converge to a limit when *D* is greater than one, but remain separated when *D* is below that limit. (Online version in colour.)

These results are quite general, but we will begin with concrete examples. We first consider the scenario in which *N*^2^ − *N* distortions are drawn randomly from some distribution *ρ*(*d*). We consider the scaling of the expected rate–distortion function over the distribution of random distortion matrices with the environmental complexity, log *N*. As argued previously [41], in the large *N* limit, the rate–distortion function of any given environment becomes arbitrarily close to this expectation value with high probability. This previously described result is apparent in simulation results in figure 1, but so is the above-mentioned presence of two regimes: when *ρ*(*d*) is a mean-shifted exponential, rate *R*(*D*) appears to grow with environmental complexity log *N* only for distortions *D* below the minimal confound *d*_min_ = 1.

**Figure 2.**
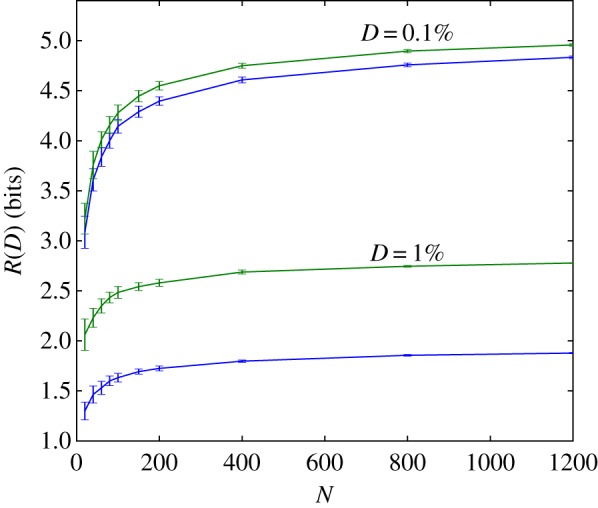
Fault-tolerant organisms can radically simplify their environment. When an organism can tolerate errors in perception, large savings in storage are possible. Shown here is the scaling between environmental size, *N*, and perceptual costs, *R*(*D*), when we constrain the average distortion, *D* to be 1% and 0.1% of the average cost of random guessing. We consider both environments with exponential and with lognormal (heavy-tailed) fitness, indicated by blue (lower of pair) and green (upper of pair), respectively; in both cases, 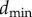 is equal to zero. A simple argument (see the text) shows that in this low-fidelity regime, and as *N* goes to infinity, perceptual demands asymptote to a constant. (Online version in colour.)

**Figure 3.**
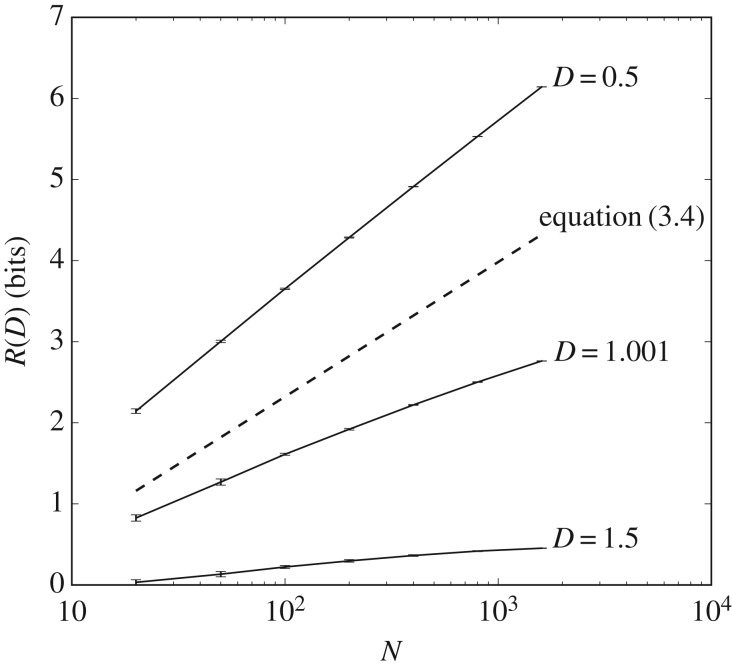
The high-fidelity regime is sensitive to environmental complexity. Shown here are encoding costs for the shifted exponential distribution, where all errors have a minimal penalty, 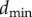, of one. When we allow average distortions to be greater than 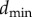, we are in the low-fidelity regime, and costs, as measured by *R*(*D*), asymptote to a constant. In the high-fidelity regime, when *D* is less than 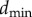, costs continue to increase, such that *R*(*D*) becomes linear in log *N*; the dashed line shows a comparison to the lower bound given in equation (3.4). The change-over in scaling happens at 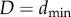. Standard errors and means are calculated from 25 samples.

Simulation results for the mean-shifted exponential and lognormal distributions *ρ*(*d*) shown in figures 2 and 3 reveal how quickly rate increases with environmental complexity. In the low-fidelity regime, the rate averaged over the distortion matrix distribution, 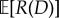, asymptotes to a finite constant as environmental complexity log *N* tends to infinity, as shown in figure 2. In the high-fidelity regime, rate *R*(*D*) scales linearly with environmental complexity log *N*, as shown in figure 3. The rate at which expected resources scales with environmental complexity increases as the desired distortion *D* decreases, appearing to slow to 0 as *D* approaches 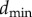.

In the low-fidelity regime, the suggestive results of the simulations can be confirmed by a simple analytic argument. For each of the *N* environmental states, and expected distortion *D*, there will be, on average, *NP*(*d* < *D*) states that are allowable ambiguities—i.e. the organism can take as synonyms for that percept. While this is not the most efficient coding, it provides an upper bound to the optimal rate of
3.1
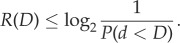
For the exponential, this upper bound means that ambiguities accumulate sufficiently fast that the organism will need at most 6.6 bits (for *D* equal to 1%) or 10 bits (for *D* equal to 0.1%) even when the set of environmental states becomes arbitrarily large.

That the bound relies on the conditional distribution function means that our results are robust to heavy-tailed distributions. What matters is the existence of harmless synonyms; for the states we end up being forbidden to confuse, penalties can be arbitrarily large. We can see this in figure 2, where we consider a lognormal distribution with mean and variance chosen so that *P*(*d* < 0.1%) is identical to the exponential case. The existence of large penalties in the lognormal case does not affect the asymptotic behaviour.

These simulation results show not only the validity of the bound, but that actual behaviour has a rapid asymptote; in both the exponential and lognormal cases, the scaling of *R*(*D*) is slower than logarithmic in *N*.

This bound is not tight; as can be seen in figure 2, far better compressions are possible, and the system asymptotes at much lower values. Even so, this bound in equation (3.1) implies that the limit of the expected rate–distortion functions in the low-fidelity regime as environmental complexity increases exists.

More generally, in structured environments, *R*(*D*) is bounded from above so that 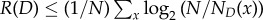, where *N*_*D*_(*x*) is the number of synonyms for *x* with distortion penalty less than *D*. As long as *N*_*D*_ is asymptotically proportional to *N*, this upper bound implies asymptotic insensitivity to the number of states of the environment.

This remarkable result has, at first, a counterintuitive feel. As environmental richness rises, it seems that the organism should have to track increasing numbers of states. However, the compression efficiency depends on the difference between environmental uncertainty before and after the signal is received, and these two differences, in the asymptotic limit, scale identically with *N*.

The existence of asymptotic bounds on memory can also be understood by an example from software engineering. As the web grows in size, a search tool such as Google needs to track an increasing number of pages (environmental complexity rises). If the growth of the web is uniform, and the tool is well built, however, the number of keywords a user needs to put into the search query does not change over time. For any particular query—‘information theory neuroscience’, say—the results returned will vary, as more and more relevant pages are created (ambiguity rises), but the user will be similarly satisfied with the results (error costs remain low).^1^

As we approach 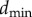, the upper bound given by log_2_(1/*P*(*d* < *D*)) becomes increasingly weak, diverging when 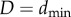. As we approach this threshold, we enter the high-fidelity regime of 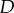, where organisms attempt to distinguish difference at a fine-grained level. In this regime, rate increases without bound as environmental complexity increases.

The apparent linear scaling of *R*(*D*) with log *N* in the high-fidelity regime shown in figure 3 is confirmed by another simple analytic argument. As in the previous case, we can upper-bound coding costs by construction of a sub-optimal codebook; we can also lower-bound coding costs by finding the optimal codebook for a strictly less-stringent environment. Here, the sub-optimal codebook allocates a total probability *C* equally to all off-diagonal elements. The rate of this codebook is log_2_ *N* − *H*_*b*_(*C*) − *C* log_2_(*N* − 1), while its expected distortion is 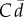, where
3.2
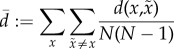
is the mean off-diagonal distortion. This yields an upper bound on the true rate–distortion function
3.3
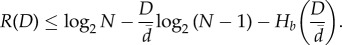
A less-stringent environment is one in which all distortions are 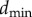, and as 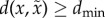, this environment has strictly lower resource costs for the same distortion. The rate–distortion function of this environment was given in [60]
3.4
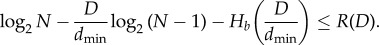
Together, these place upper and lower bounds on the true rate–distortion function in the high-fidelity regime. In the large *N* limit, these bounds simplify to
3.5
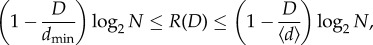
plus small *O*(1) corrections. In short, the rate scales as *O*(log*N*) in the high-fidelity regime, 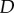, as expected.

**Figure 4.**
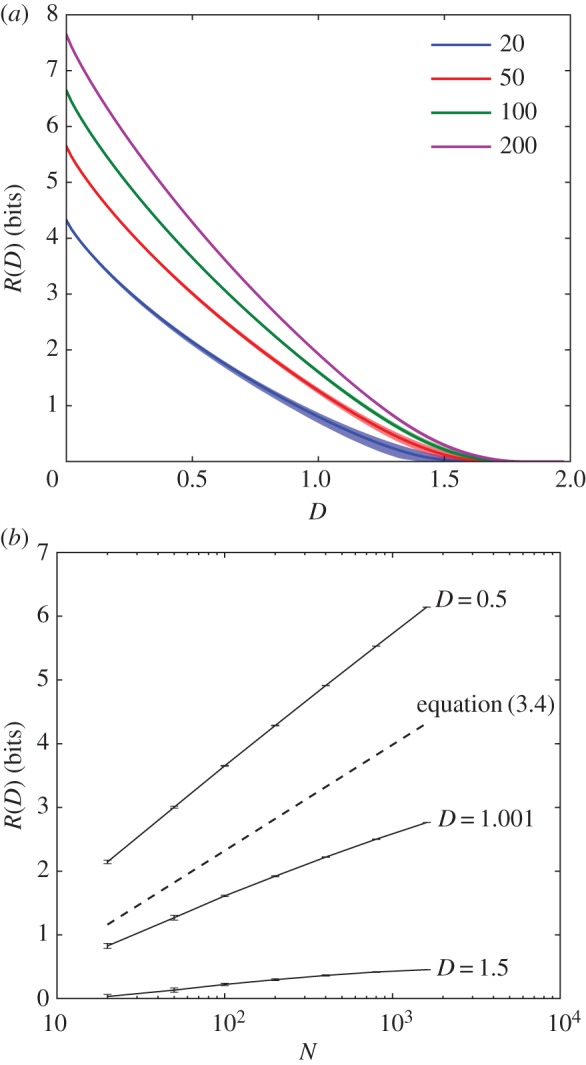
Scaling results for a more structured environment. The distortion matrices are drawn randomly as described in the main text. (*a*) The rate–distortion function with shaded 68% confidence intervals and mean 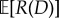 calculated from bootstrapping the rate–distortion functions of 25 different such environments. (*b*) The scaling of *R*(*D*) at various *D* and *N* for environments, with means and standard errors calculated from 25 samples. (Online version in colour.)

These scaling results hold even when off-diagonal entries are partially correlated, as long as correlation scales grow more slowly than environment size so that *N*_*D*_ ∝ *N*. As a step towards introducing more structure into these environments, we consider drawing distortion matrices as follows. First, an initial *N* × *N* distortion matrix is constructed by drawing 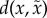 i.i.d. from a mean-shifted exponential, 
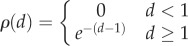
. Next, each entry 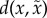 is replaced by 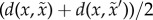, where 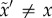 is randomly chosen. This final distortion matrix has pairwise-correlated entries. Aforementioned scaling results hold, both using the analytic arguments above and simulation results in figure 4.

**Figure 5.**
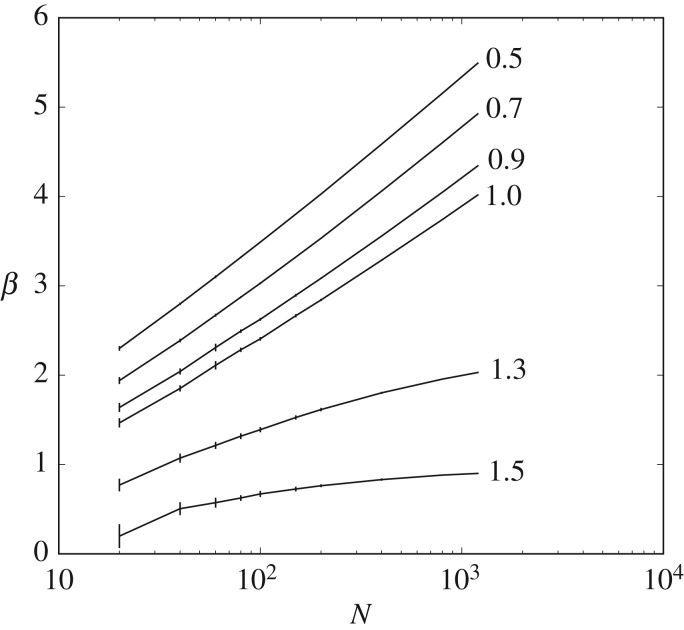
The metabolic efficiency required to achieve a particular distortion in the high-fidelity regime increases with environmental complexity. Shown are plots of the metabolic efficiency, *β*, required to achieve a particular distortion as a function of environmental complexity, log *N*, in random environments in which 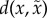 is drawn i.i.d. from 
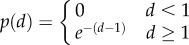
. In the high-fidelity regime, *D* < 1, the required *β* increases linearly with log *N*; in the low-fidelity regime, the required *β* asymptotes to a finite constant. As earlier, means and standard errors were calculated from 25 samples.

Finally, the gain *β* will at least need to scale as log *N* in order to retain *D* below 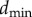, as shown in figure 5. A non-coding codebook will have an expected distortion of roughly 〈*d*〉 and a rate of 0; a codebook in the high-fidelity regime will have some expected distortion 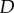 and a rate of *C*log_2_ *N* for some constant *C* bounded by 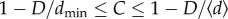. The high-fidelity codebook outperforms the non-coding codebook when *β*〈*d*〉 ≥ *βD* + *C* log_2_ *N* or when *β* ≥ (*C*/(〈*d*〉 − *D*))log_2_ *N*.

On evolutionary timescales, we expect metabolic efficiency to increase. This is seen in the long-term evolution experiments on *Escherichia coli* as it adapts to new environments [61,62], more generally, it is seen in the macroevolutionary record as the metabolic rate per gram decreases with body size while (in general) body size tends to increase [63].

In the framework described here, the effect of increasing metabolic efficiency is to increase *β* and thereby to allow the organism to achieve smaller *D*. When *N* is large and rising on timescales faster than those on which evolution increases *β*, we expect organisms to stall out at 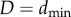. This is because whenever they go below this error rate, increases in environmental complexity *N* erase the gains. Even when they are poised around 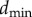, increasing *β* will still give an individual an evolutionary advantage; the effect of a changing environment is to force organisms to compete on efficiency, rather than richness of internal representations. These results imply that when organisms compete in an environment of rising complexity, we expect to find their perceptual apparatus poised around 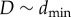. We refer to this as poised perception. Figure 5 shows visually how this driving force to 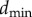 occurs when *β* rises more slowly than log(*N*).

It is useful to relate this poised perception result to the underlying rate–distortion paradigm. Rate–distortion itself can be understood as saying ‘given a particular error tolerance we want to meet, how low can the transmission rate be’. In that sense, the theory can be understood as talking about achieving a ‘just adequate’ level of processing given constraints.

Our finding on poised perception adds a second level of ‘just adequacy’. It says that an organism's error tolerance will be driven, by evolutionary forces, to a point just adequate for distinguishing minimal confounds. At that point, the organism then devotes perceptual resources ‘just adequate’ to achieving that ‘just adequate’ level of error.

## 4. Discussion

There are costs and benefits to improved sensory perception. Better tracking of the environment help organisms to acquire new resources. However, the increases in perceptual accuracy needed to achieve this tracking usually require increases in the required size or energy consumption of sensory apparatus. Our work here establishes lower bounds on these trade-offs in terms of the rate–distortion function, which describe the minimal rate required to achieve a given minimal level of perceptual inaccuracy.

We argued that these functions showed surprising regularities in large environments, even though the optimal rate-limited sensory codebooks were highly environment-dependent. Marzen & DeDeo [41] focused on the insensitivity of the rate–distortion function to particular choices of the distortion measure; here, we focus on the scaling of the rate–distortion function with environmental complexity.

Every ensemble of environments has a ‘minimal confound’ 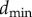, the minimal price that one pays for confusing one environmental state with another. In a low-fidelity regime, when an organism's average distortion is larger than 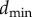, increasing environmental complexity does not increase perceptual load. As the number of environmental states increases, innocuous synonyms accumulate. They do so sufficiently fast that an organism can continue to represent the fitness-relevant features within constant memory.

It is only in a high-fidelity regime, when an organism attempts to achieve average distortions below 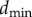, that perceptual load becomes sensitive to complexity. High-fidelity representations of the world do not scale, and an organism that attempts to break this threshold will find that, when the number of environmental states increases, it will be driven back to the low-fidelity regime, unless its perceptual apparatus rapidly increases in size.

Together, these results suggest that well-adapted organisms will evolve to a point of ‘poised perception’, where they can barely distinguish objects that are maximally similar.

It is worth considering how this analysis generalizes to the case of a continuum of environmental states. In this case, we have some (usually continuous function) 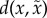. To see how this alters the analysis, let us take (as a simple choice) *d* to be mean squared error, 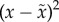. If we assume, again for specificity, that the signal itself is Gaussian with variance *σ*^2^, then an analytic calculation shows that *R*(*D*) is equal to to 
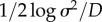
 when *D* is less than *σ*^2^ (and zero otherwise). In this case, as long as we track some information about the environment, we are (as expected) never in the ‘low-fidelity’ regime; an increase in environmental complexity (here corresponding to an increase in the range of values the environment can take on, i.e. an increase in *σ*^2^) always leads to an increase in *R*(*D*) for fixed *D*. It is only upon introducing discrete structure in the distortion matrix (e.g. the need to determine both stimulus amplitude, and stimulus type) that the separation between high- and low-fidelity regimes returns.

We have focused on the effect of increasing perceptual abilities due to changes in metabolic efficiency over evolutionary time. However, it is well-known that individuals can increase their perceptual abilities through training in ontogenetic time, as is seen, for example, in video-game players [64–67]. We expect, therefore, the emergence of poised perception on much more rapid timescales. Sims [30] has already demonstrated the utility of rate–distortion theory for the study of perceptual performance under laboratory conditions, and this suggests the possibility of testing the emergence of this phenomenon under a combination of both training (to increase *β*) and stimulus complexity *N*.

In other words, when we attempt to make distinctions within a sub-category of increasing size, we will be barely competent at making the least important distinctions. Informally, we can just about distinguish our least-important friends when our circles expand. Such ideas might be tested in a laboratory-based study that trains subjects on a set of increasingly difficult distinction tasks with varying rewards. The results here predict that, as they improve their decision-making abilities, individuals will develop representations that are barely able to make distinctions between the least-important cases. It is also possible to consider the simulation of artificial environments, in an evolutionary robotics or artificial life paradigm [68], to determine the parameter ranges (selection pressure, growth rate of complexity) under which these bounds are achieved.

In many cases, the threats and benefits posed by an environment have a hierarchical structure: there are many threats that are roughly equally bad, and there are many forms of prey, say, that are roughly equally good. Such a hierarchical structure means that we do not expect organisms to have a single system for representing their environment. The results here then apply to sub-domains of the perceptual problem. In the case of predator–prey, for example, we expect optimal codebooks will look block-diagonal, with a clear predator–prey distinction, and then sub-distinctions for the two categories (eagle versus leopard; inedible versus edible plants).

Similar implications apply in the cognitive and social sciences, where the semantics may play a role in organizing a perceptual hierarchy. A small number of coarse-grained distinctions are often found in human social cognition, where we expect a block-diagonal structure over a small, fixed number of categories (kin versus non-kin, in-group versus out-group [69]). Within each of the large distinctions, our representational systems are then tasked with making fine-grained distinctions over sets of varying size.

For the large distinction, *N* is fixed, but environmental complexity can increase within each category. As new forms of predators or prey arise, the arguments here suggest that organisms will be poised at the thresholds within each subsystem. When, for example, prey may be toxic, predators will evolve to distinguish toxic from non-toxic prey, but tend not to distinguish between near-synonyms *within* either the toxic or non-toxic categories.

One example of the role played by poised perception in the wild is the evolution of Batesian mimicry—where a non-toxic prey species imitates a toxic one—when such mimicry is driven by the perceptual abilities of predators [70]. Our arguments here suggest that predator perception evolves in such a way that Batesian mimics will have a phenotypic range similar to that found between two toxic species. A harmless Batesian mimic can then emerge if it can approximate, in appearance, a toxic species by roughly the same amount as that species resembles a second species, also toxic. This also suggests that a diversity of equally toxic species will lead to less-accurate mimicry. This is not because a mimic will attempt to model different species simultaneously (the multimodal hypothesis [71]), rather that predators who attempt to make finer-grained distinctions within the toxic-species space will find themselves driven back to 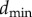 when the total number of species increases.

This work is only a first step towards a better understanding of trade-offs between accuracy and perceptual cost in biological systems. Most notably, by reference to a look-up table, or codebook, we neglect the costs of processing the information. The challenges of post-processing, or storing, the output of a perceptual module may argue for adding additional constraints to equation (2.4); see, e.g. [72], where constraints on memory of the input suggest the use of deterministic codebooks, and the more general discussion of the varieties of computation costs in [14].

## 5. Conclusion

In any environment, there will be some levels of perceptual accuracy that are nearly impossible for any physical being to achieve. Outside of those effectively forbidden regions, the required size of the sensory apparatus may well depend only on very coarse environmental statistics [41]. Well-adapted organisms are then likely to take advantage of such regularities, allocating resources to gain the information they need to survive.

When they do this, they will encounter not only the constraints expected from the material conditions they experience in both ontogenetic and evolutionary time, but also constraints that arise from the fact that they are, in part, simply information processors in a stochastic but regular world. Our findings suggest that the challenges of perception dramatically increase when organisms attempt to enter a high-fidelity regime. Organisms that are ‘too good’ at perception will find that their abilities rapidly erode when environmental complexity increases, driving them to transition point back into a lower-fidelity regime.

## Endnote

1 We are grateful to Michael Lachmann for suggesting this example.

## Data accessibility

The code necessary to reproduce the figures in this paper has been released as the Python package, PyRated, and is available at http://bit.ly/pyrated.
